# Supplementary material for: Optimization of Production Parameters for Probiotic Lactobacillus Strains as Feed Additive
Source: Molecules. 2019 Sep 9;24(18):3286. doi: 10.3390/molecules24183286 (PMC6767249; doi:10.3390/molecules24183286)
Supplement: Supplementary file 1 [file molecules-24-03286-s001.zip › supplementary materials.docx]

**Supplementary information**

Supplementary Table 1: Actual and coded levels of variables employed in the Box-Bohnken design

| Individual variables | | Coded values | | |
| --- | --- | --- | --- | --- |
|  |  | -1 | 0 | 1 |
| Sucrose (g/mL) | X_1_ | 0.05 | 0.075 | 0.10 |
| Skim milk (g/mL) | X_2_ | 0.075 | 0.1125 | 0.15 |
| Trehalose (g/mL) | X_3_ | 0.05 | 0.075 | 0.10 |

Supplementary Table 2: Factors and responses of the Box-Behnken design (BBD)

| Runs | Factors | | | Response *L. salivarius* | | Response *L. agilis* | |
| --- | --- | --- | --- | --- | --- | --- | --- |
|  | X_1_: Skim milk | X_2_: Sucrose | X_3_: Trehalose | Actual response | Predicted response | Actual response | Predicted response |
| 1 | 0 | 1 | 1 | 63.41 | 64.50 | 71.42 | 71.45 |
| 2 | -1 | 0 | 1 | 57.29 | 57.03 | 67.53 | 67.25 |
| 3 | 0 | -1 | 1 | 47.13 | 46.77 | 68.20 | 67.20 |
| 4 | 0 | 0 | 0 | 72.42 | 72.90 | 77.01 | 77.26 |
| 5 | 0 | 0 | 0 | 72.71 | 72.90 | 76.85 | 77.26 |
| 6 | 0 | 1 | -1 | 58.74 | 59.10 | 67.84 | 68.84 |
| 7 | 1 | 1 | 0 | 73.91 | 73.29 | 81.44 | 80.16 |
| 8 | 0 | 0 | 0 | 73.04 | 72.90 | 76.98 | 77.26 |
| 9 | 1 | -1 | 0 | 51.58 | 52.41 | 77.62 | 77.37 |
| 10 | 1 | 0 | -1 | 68.87 | 69.13 | 79.43 | 79.71 |
| 11 | 0 | -1 | -1 | 50.89 | 49.80 | 60.37 | 60.33 |
| 12 | 0 | 0 | 0 | 73.10 | 72.90 | 77.48 | 77.26 |
| 13 | -1 | 1 | 0 | 53.47 | 52.64 | 66.32 | 66.56 |
| 14 | -1 | -1 | 0 | 45.88 | 46.50 | 55.32 | 56.59 |
| 15 | -1 | 0 | -1 | 55.87 | 56.34 | 60.81 | 59.57 |
| 16 | 0 | 0 | 0 | 73.23 | 72.90 | 77.96 | 77.26 |
| 17 | 1 | 0 | 1 | 71.27 | 70.80 | 80.27 | 81.51 |


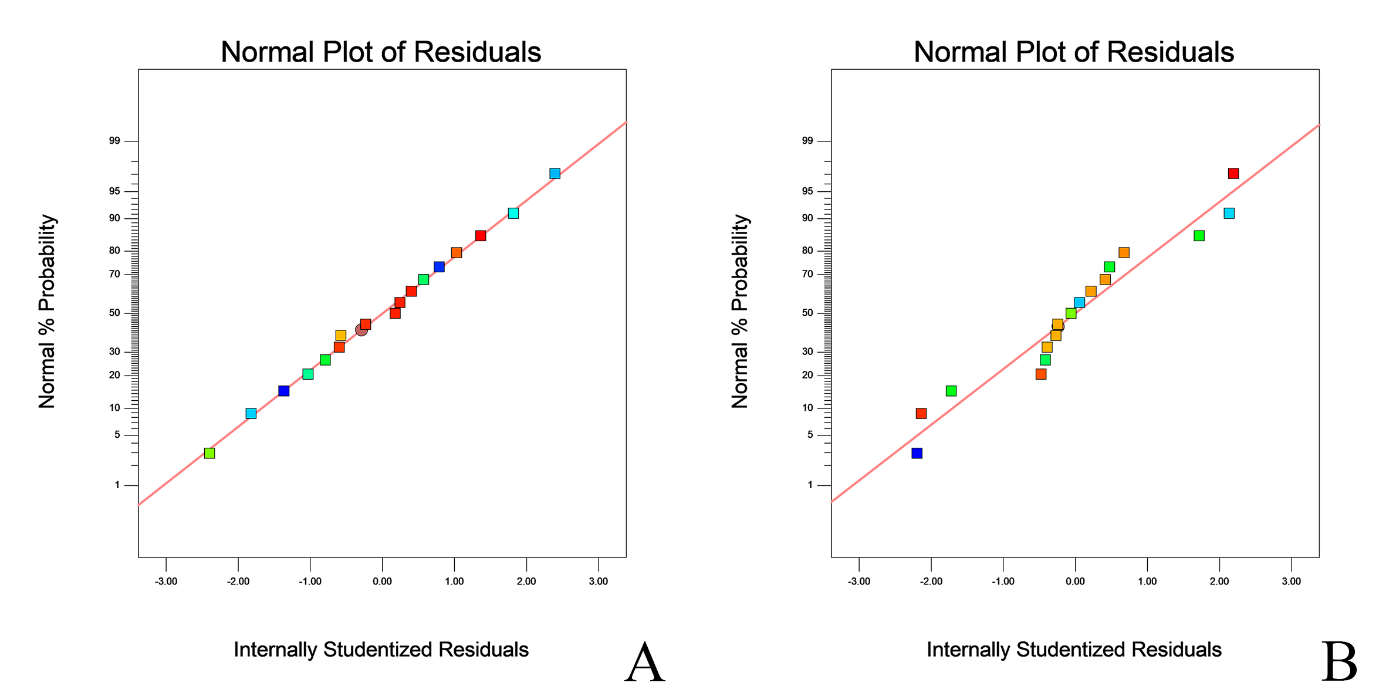


Supplementary Figure 1: Linear plot fitting normal plot of residuals. A: *L. salivarius*, B: *L. agilis*

Supplementary Table 3: Composition of feed used for in-feed storage experiments

| Ingredients | Basal diet [as fed, %] |
| --- | --- |
| Maize | 32.03 |
| Wheat | 24.78 |
| Soybean meal 49 % CP | 32.33 |
| soybean oil | 5.95 |
| Mineral-Vitamin Premix | 1.2 |
| Limestone | 1.46 |
| Monocalcium phosphate | 1.84 |
| Salt | 0.1 |
| Methionin | 0.18 |
| Lysin HCL | 0.13 |
