# Supplementary material for: Optimization of Production Parameters for Probiotic Lactobacillus Strains as Feed Additive
Source: Molecules. 2019 Sep 9;24(18):3286. doi: 10.3390/molecules24183286 (PMC6767249; doi:10.3390/molecules24183286)
Supplement: Supplementary file 1 [file molecules-24-03286-s001.zip › supplementary materials/Supplementary Table 1.docx]

Supplementary Table 1: Actual and coded levels of variables employed in the Box-Bohnken design

| Individual variables | | Coded values | | |
| --- | --- | --- | --- | --- |
|  |  | -1 | 0 | 1 |
| Sucrose (g/mL) | X_1_ | 0.05 | 0.075 | 0.10 |
| Skim milk (g/mL) | X_2_ | 0.075 | 0.1125 | 0.15 |
| Trehalose (g/mL) | X_3_ | 0.05 | 0.075 | 0.10 |
