# Supplementary material for: Optimization of Production Parameters for Probiotic Lactobacillus Strains as Feed Additive
Source: Molecules. 2019 Sep 9;24(18):3286. doi: 10.3390/molecules24183286 (PMC6767249; doi:10.3390/molecules24183286)
Supplement: Supplementary file 1 [file molecules-24-03286-s001.zip › supplementary materials/Supplementary Table 2.docx]

Supplementary Table 2: Factors and responses of the Box-Behnken design (BBD)

| Runs | Factors | | | Response *L. salivarius* | | Response *L. agilis* | |
| --- | --- | --- | --- | --- | --- | --- | --- |
|  | X_1_: Skim milk | X_2_: Sucrose | X_3_: Trehalose | Actual response | Predicted response | Actual response | Predicted response |
| 1 | 0 | 1 | 1 | 63.41 | 64.50 | 71.42 | 71.45 |
| 2 | -1 | 0 | 1 | 57.29 | 57.03 | 67.53 | 67.25 |
| 3 | 0 | -1 | 1 | 47.13 | 46.77 | 68.20 | 67.20 |
| 4 | 0 | 0 | 0 | 72.42 | 72.90 | 77.01 | 77.26 |
| 5 | 0 | 0 | 0 | 72.71 | 72.90 | 76.85 | 77.26 |
| 6 | 0 | 1 | -1 | 58.74 | 59.10 | 67.84 | 68.84 |
| 7 | 1 | 1 | 0 | 73.91 | 73.29 | 81.44 | 80.16 |
| 8 | 0 | 0 | 0 | 73.04 | 72.90 | 76.98 | 77.26 |
| 9 | 1 | -1 | 0 | 51.58 | 52.41 | 77.62 | 77.37 |
| 10 | 1 | 0 | -1 | 68.87 | 69.13 | 79.43 | 79.71 |
| 11 | 0 | -1 | -1 | 50.89 | 49.80 | 60.37 | 60.33 |
| 12 | 0 | 0 | 0 | 73.10 | 72.90 | 77.48 | 77.26 |
| 13 | -1 | 1 | 0 | 53.47 | 52.64 | 66.32 | 66.56 |
| 14 | -1 | -1 | 0 | 45.88 | 46.50 | 55.32 | 56.59 |
| 15 | -1 | 0 | -1 | 55.87 | 56.34 | 60.81 | 59.57 |
| 16 | 0 | 0 | 0 | 73.23 | 72.90 | 77.96 | 77.26 |
| 17 | 1 | 0 | 1 | 71.27 | 70.80 | 80.27 | 81.51 |
