# Supplementary material for: Optimization of Production Parameters for Probiotic Lactobacillus Strains as Feed Additive
Source: Molecules. 2019 Sep 9;24(18):3286. doi: 10.3390/molecules24183286 (PMC6767249; doi:10.3390/molecules24183286)
Supplement: Supplementary file 1 [file molecules-24-03286-s001.zip › supplementary materials/Supplementary Table 3.docx]

Supplementary Table 3: Composition of feed used for in-feed storage experiments

| Ingredients | Basal diet [as fed, %] |
| --- | --- |
| Maize | 32.03 |
| Wheat | 24.78 |
| Soybean meal 49 % CP | 32.33 |
| soybean oil | 5.95 |
| Mineral-Vitamin Premix | 1.2 |
| Limestone | 1.46 |
| Monocalcium phosphate | 1.84 |
| Salt | 0.1 |
| Methionin | 0.18 |
| Lysin HCL | 0.13 |
