# Supplementary figures and images for: Optimization of Production Parameters for Probiotic Lactobacillus Strains as Feed Additive
Source: Molecules. 2019 Sep 9;24(18):3286. doi: 10.3390/molecules24183286 (PMC6767249; doi:10.3390/molecules24183286)

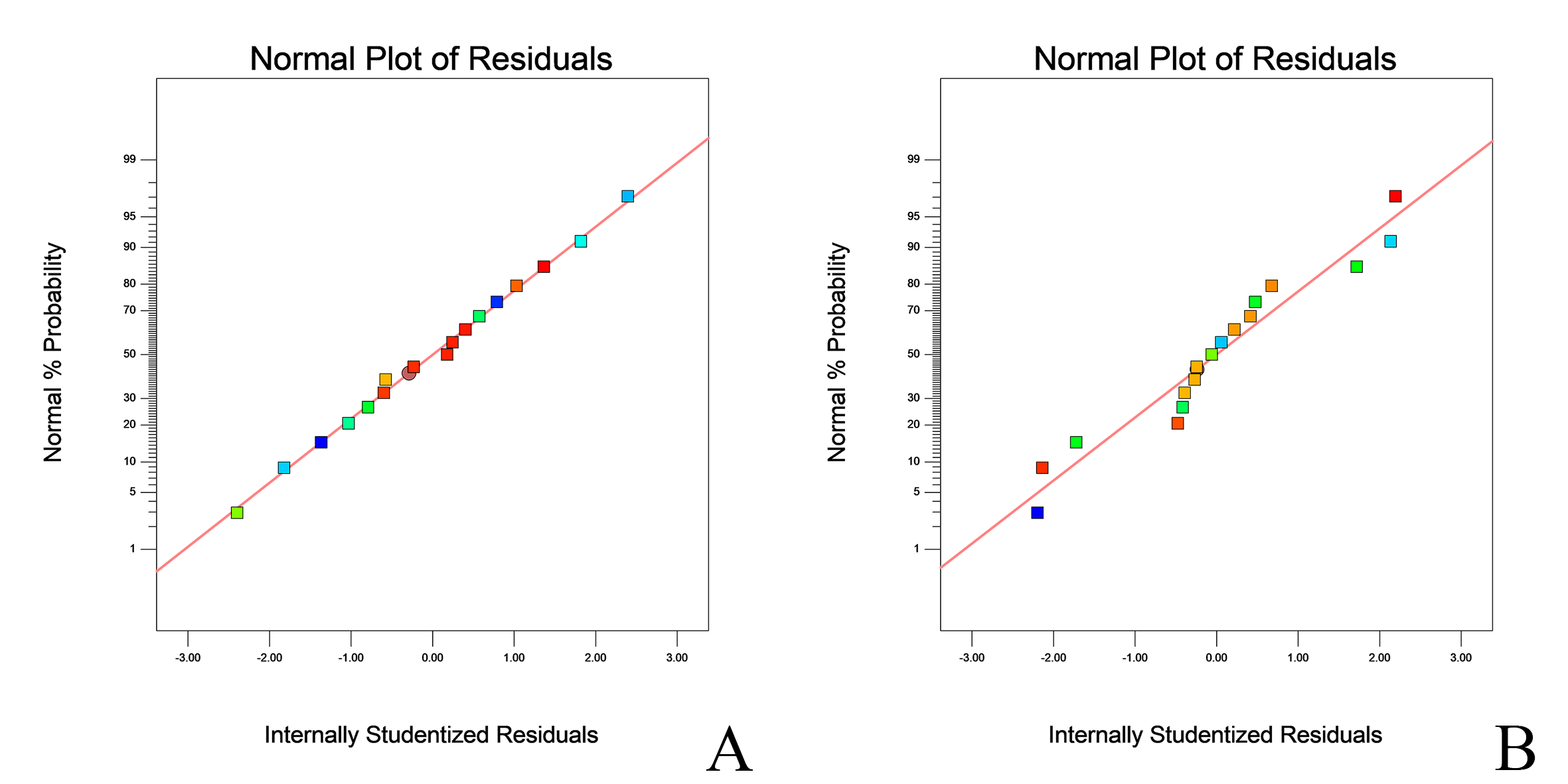

Supplement: Supplementary file 1 [file molecules-24-03286-s001.zip › supplementary materials/Supplementary Figure 1 Linear plot fitting normal plot of residuals. A L. salivarius, B L. agilis.tif]
